# Supplementary material for: LKB1 regulates JNK-dependent stress signaling and apoptotic dependency of KRAS-mutant lung cancers
Source: Nat Commun. 2025 May 2;16:4112. doi: 10.1038/s41467-025-58753-y (PMC12048556; doi:10.1038/s41467-025-58753-y)
Supplement: Supplementary file 1 — Supplementary Information [file 41467_2025_58753_MOESM1_ESM.pdf]

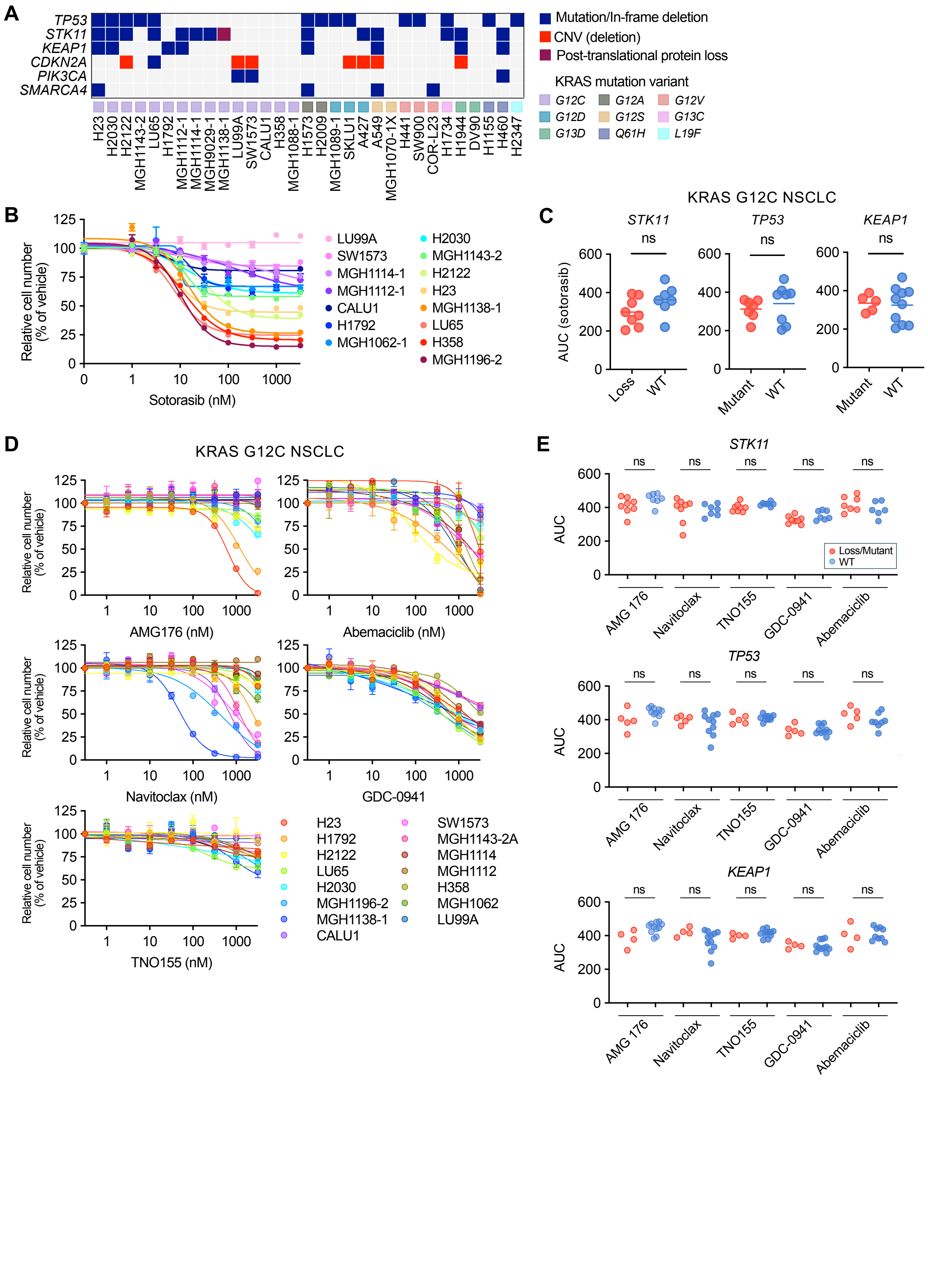

Supplementary Figure 1

Supplementary Figure 1. Sensitivity of *KRAS*^{G12C}-mutant NSCLC cell lines to sotorasib and trametinib drug combinations. **A.** Major co-occurring mutations in *KRAS*-mutant cell lines used in this study. **B.** Sotorasib sensitivity of *KRAS*^{G12C}-mutant NSCLC cell lines. Cells were treated with sotorasib for 3 days and viability was determined by CellTiter-Glo (CTG). **C.** Comparison of sotorasib sensitivity (quantified by AUC of sotorasib dose response curve) of *KRAS*^{G12C}-mutant cell lines grouped by co-occurring mutations. Each dot represents the mean AUC of 3 independent biological replicates. **D.** Drug sensitivity of *KRAS*^{G12C}-mutant NSCLC cell lines to AMG 176, navitoclax, TNO 155, abemaciclib and GDC-0941. Cells were treated with each drug for 3 days and viability was determined by CTG. Each dot represents mean and S.E.M. **E.** Comparison of sensitivities of AMG 176, navitoclax, TNO 155, abemaciclib and GDC-0941 (quantified by AUC of dose response curve) of *KRAS*^{G12C}-mutant cell lines grouped by co-occurring mutations. Source data are provided as a Source Data file.

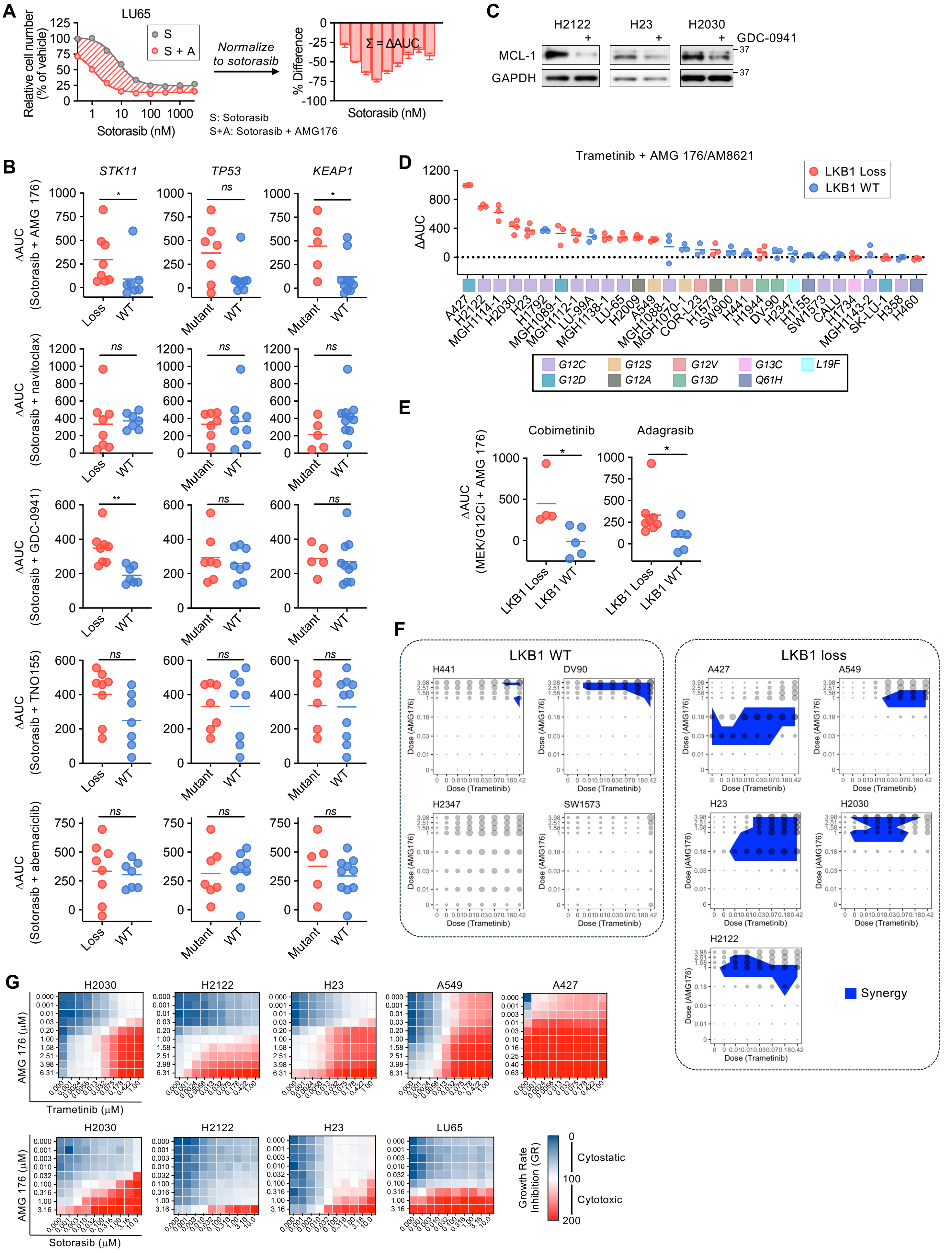

Supplementary Figure 2

Supplementary Figure 2. Inhibition of MCL-1 is synergistic with MAPK inhibition in cell lines with loss of LKB1 that exhibit high apoptotic responses. A. Calculation of ΔAUC as a metric of dependence on targeted alternate pathway in the presence of suppression of KRAS or MEK signaling. For instance, MCL-1 dependence as determined by relative sensitivity to sotorasib + AMG 176 compared to sotorasib alone. Upper left panel is duplicated from Figure 1C for comparison purposes. **B.** Cells were treated with sotorasib or sotorasib + AMG176/ navitoclax/ GDC-0941/ TNO155/abemaciclib for 3 days and viability was determined by CTG. ΔAUC was calculated as described in panel A. *STK11* loss correlates with higher ΔAUC to sotorasib + AMG 176 and sotorasib + GDC-0941 in $KRAS^{G12C}$-mutant NSCLC cell lines. *STK11*: *p=0.029, *KEAP1*: *p=0.04, unpaired-nonparametric t test, 2-sided. **C.** MCL-1 down-regulation in response to PI3K inhibition. Cells were treated with 1 μM GDC-0941 for 24 hours and MCL-1 protein levels were analyzed by western blotting. **D.** ΔAUC of trametinib + AMG 176 (or the related compound AM-8621) versus trametinib alone. Each dot represents an independent biological replicate (N=3-4). **E.** LKB1-deficient cell lines are similarly sensitive to cobimetinib (MEKi) or adagrasib (KRAS G12Ci) combined with AMG 176. Adagrasib: *p=0.02, Cobimetinib: *p=0.016, unpaired-nonparametric t test, 2-sided. **F.** Synergistic interaction between trametinib and AMG 176 in LKB1-loss cell lines. $KRAS^{G12C}$-mutant NSCLC cell lines were treated with increasing dose of trametinib and AMG 176 for 3 days and cell viability was determined by CTG. Synergy was calculated by the generalized Loewe method (see Method). Data are averaged from 3 independent biological replicates for each cell line. **G.** Cytotoxicity was observed in *LKB1*-deficient $KRAS^{G12C}$-mutant NSCLC cell lines with combined MAPK + MCL-1 inhibition. $KRAS^{G12C}$-mutant NSCLC cell lines were treated with increasing doses of trametinib/sotorasib + AMG 176 for 3 days and viability was determined by CTG. Growth Rate Inhibition was calculated by normalizing to vehicle treated cells (0), day 0 cell viability (100), and no viable cells (200) using the same data as synergy calculation in Panel A. Source data are provided as a Source Data file.

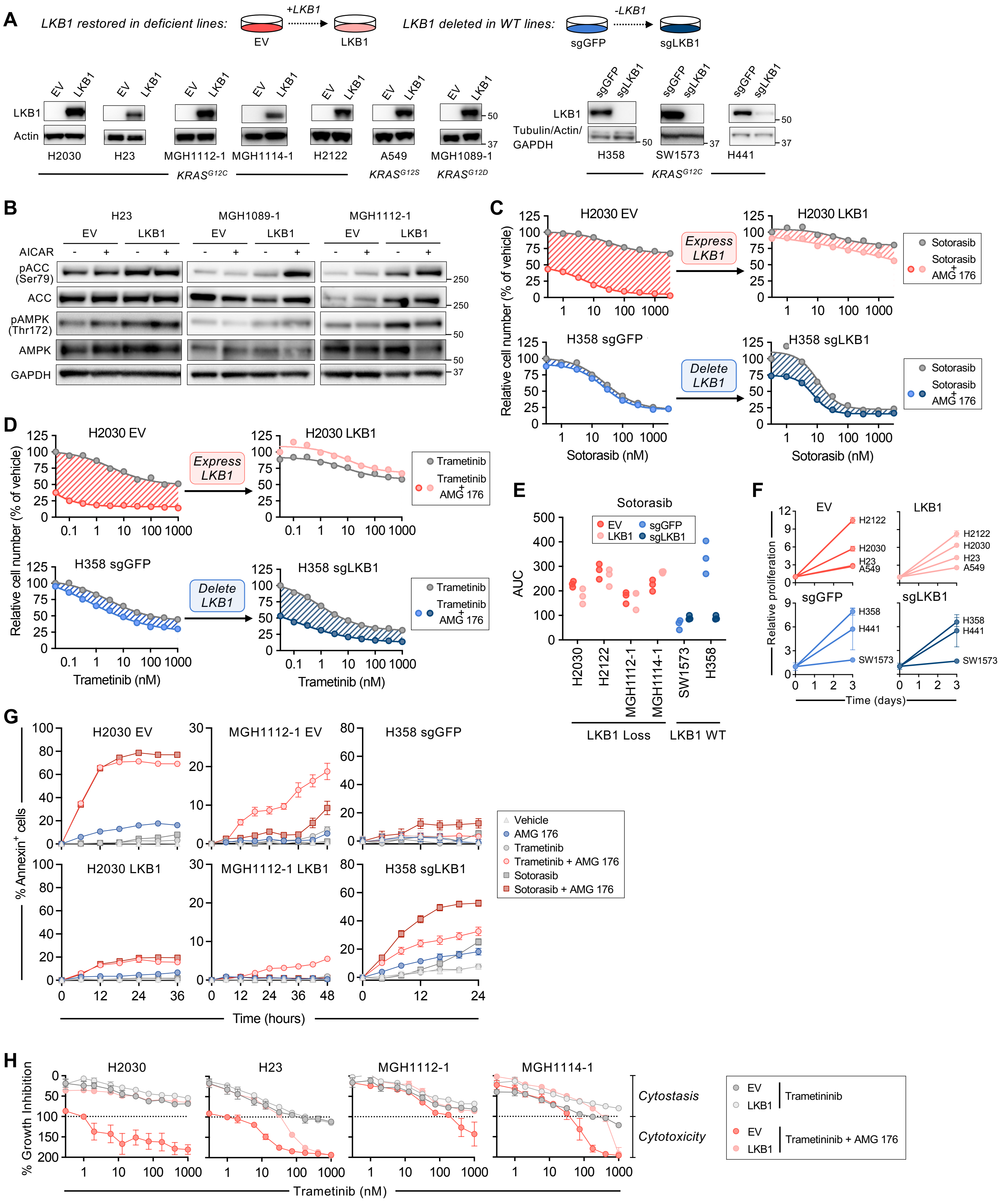

Supplementary Figure 3

Supplementary Figure 3. LKB1-deficient cells have increased sensitivity to combined MAPK + MCL-1 inhibition. A. Left: Expression of LKB1 in LKB1-deficient *KRAS*-mutant NSCLC cell lines. (EV = empty vector). Right: CRISPR-mediated knockout of LKB1 in LKB1-deficient *KRAS*-mutant NSCLC cell lines. **B.** Western blot assessment of pAMPK (T172) and pACC (S79) in H23, MGH1089-1 and MGH1112-1 EV and LKB1 cells after treatment with 0.5 mM AICAR for 6 hours. **C-D.** Dose response curves for H2030 EV, H2030 LKB1, H358sgGFP (Crispr KO of GFP), and H358sgLKB1 (Crispr KO of LKB1) cell lines after treatment with increasing doses of trametinib/sotorasib in the absence or presence of 1 μM of AMG 176. Data is representative of biological replicates (N=2, 4). **E.** Re-expression of LKB1 in LKB1-deficient cells, or LKB1 deletion in LKB1 WT cells, does not alter sensitivity to sotorasib alone (expressed as AUC of sotorasib dose response curve). Each dot represents an independent biological replicate. **F.** Relative proliferation of isogenic cell line pairs. Each dot represents an independent biological replicate. **G.** Restoration of LKB1 in LKB1-deficient *KRAS*-mutant cell lines, or LKB1 deletion in LKB1 WT cells, increases apoptotic response (annexin + cells) to trametinib + AMG 176, assessed by live cell imaging. Data are mean and S.E.M. of 3 technical replicates. Veh and Sotorasib + AMG 176 data from Figure 2D are replotted here for comparison purposes. **H.** Cytotoxic versus cytostatic response as determined by calculating Growth Rate Inhibition (vehicle treated cells = 0, day 0 cell viability = 100, no viable cells = 200). Cells were treated for 3 days and viability determined by CTG. Data are mean and S.E.M. from 3 independent biological replicates. Source data are provided as a Source Data file. Representative data for western blots has been replicated at least 2 times.

A

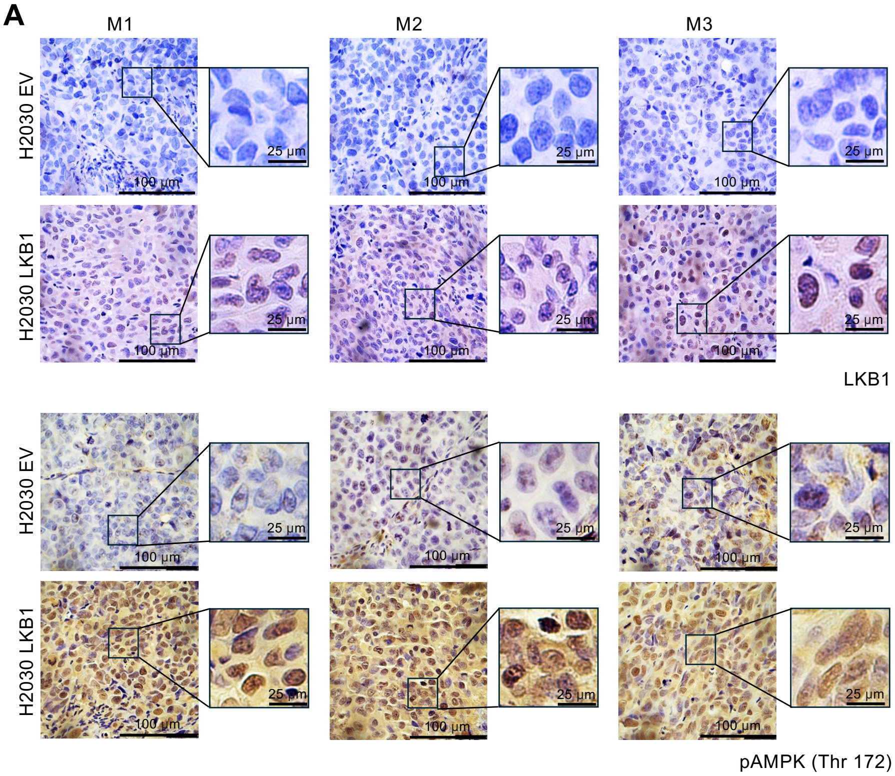

B

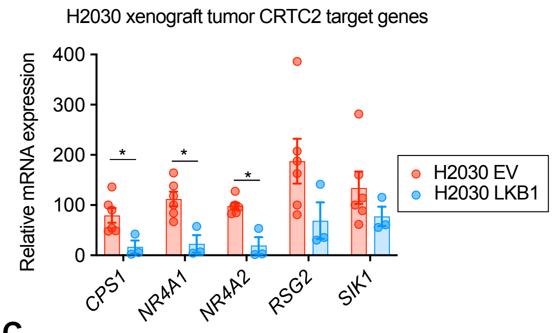

C

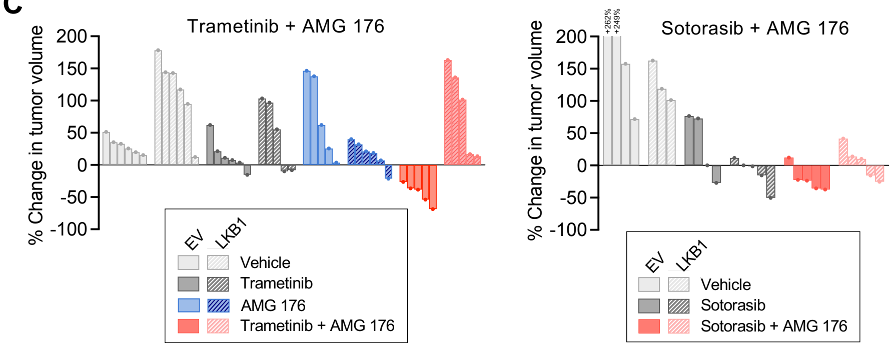

Supplementary Figure 4

Supplementary Figure 4. Characteristics of H2030 isogenic xenograft tumors. A. Representative immunohistochemical staining of LKB1 and pAMPK (T172) in H2030 EV (empty vector) and LKB1 xenograft tumors. **B.** qRT-PCR of CRTC/CREB target genes in H2030 EV and LKB1 xenograft tumors. Each dot represents an independent mouse tumor (N=3,6, $^*p=0.024$, unpaired-nonparametric t test, 2-sided). **C.** Waterfall plots showing degree of tumor volume change of H2030 isogenic xenograft tumors after treatment with sotorasib (30 mg/kg daily), trametinib (3 mg/kg daily), or in combination with AMG 176 (50 mg/kg daily) for 3.5 weeks. Source data are provided as a Source Data file. Western blots and immunoprecipitation images are representative of at least 2 independent biological replicates.

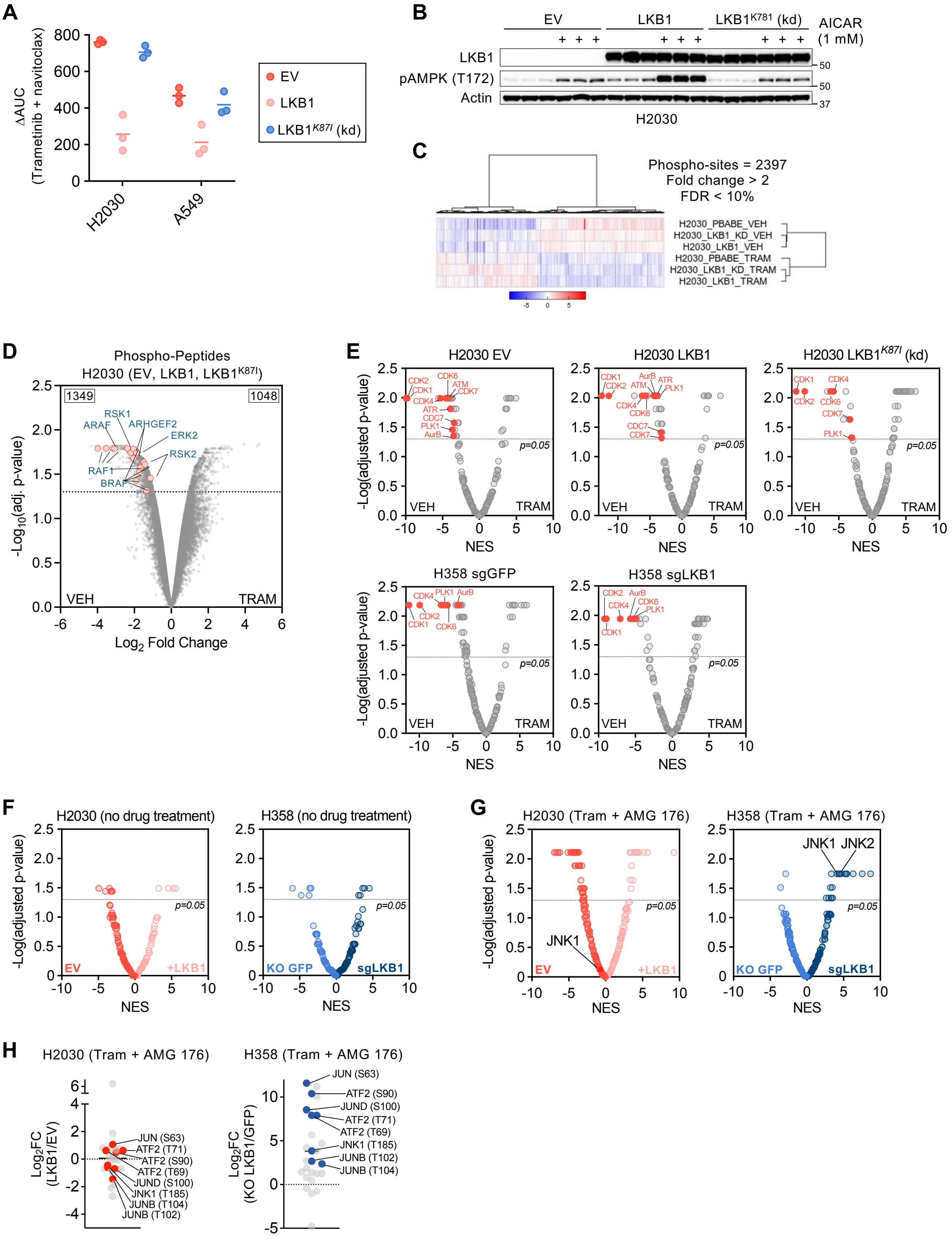

Supplementary Figure 5

Supplementary Figure 5. Phosphoproteomic analysis reveals JNK activation in LKB1-deficient *KRAS*-mutant cells after drug treatment. A. Quantification of ΔAUC from CTG assays of *KRAS*-mutant NSCLC cell lines with LKB1 loss (EV), LKB1 restoration (LKB1), or restoration of a kinase dead version of LKB1 (LKB1^{K87I} kd) after treatment with trametinib + 1 µM AMG 176. Each dot represents an independent biological replicate (N=3). **B.** Western blot assessment of LKB1 and pAMPK (T172) in H2030 EV, H2030 LKB1, and H2030 LKB1 kd cells after treatment with 1 mM AICAR for 8 hours. The H2030 panel of Figure S3A is duplicated here for comparison purposes. **C-D.** Differential levels of phosphopeptides between trametinib and vehicle treatment in H2030 EV, H2030 LKB1, and H2030 LKB1 kd cell lines (grouped). Number of differentially quantified phosphopeptide and proteins with fold-change > 2-fold and adjusted p value < 0.05 are indicated. **E.** Volcano plots showing differentially enriched signatures between trametinib and vehicle treatment. Phosphopeptide signatures were calculated using ssGSEA2.0/PTM-SEA. **F.** Differentially enriched phosphopeptide signatures in paired isogenic cell lines in the absence of drug treatment. **G.** Differential enrichment of phosphopeptide signatures in trametinib + AMG 176-treated isogenic cell line pairs. **H.** Differential phosphorylation of individual JNK substrates in trametinib + AMG 176 treated isogenic cell line pairs. TRAM – trametinib. Source data are provided as a Source Data file.

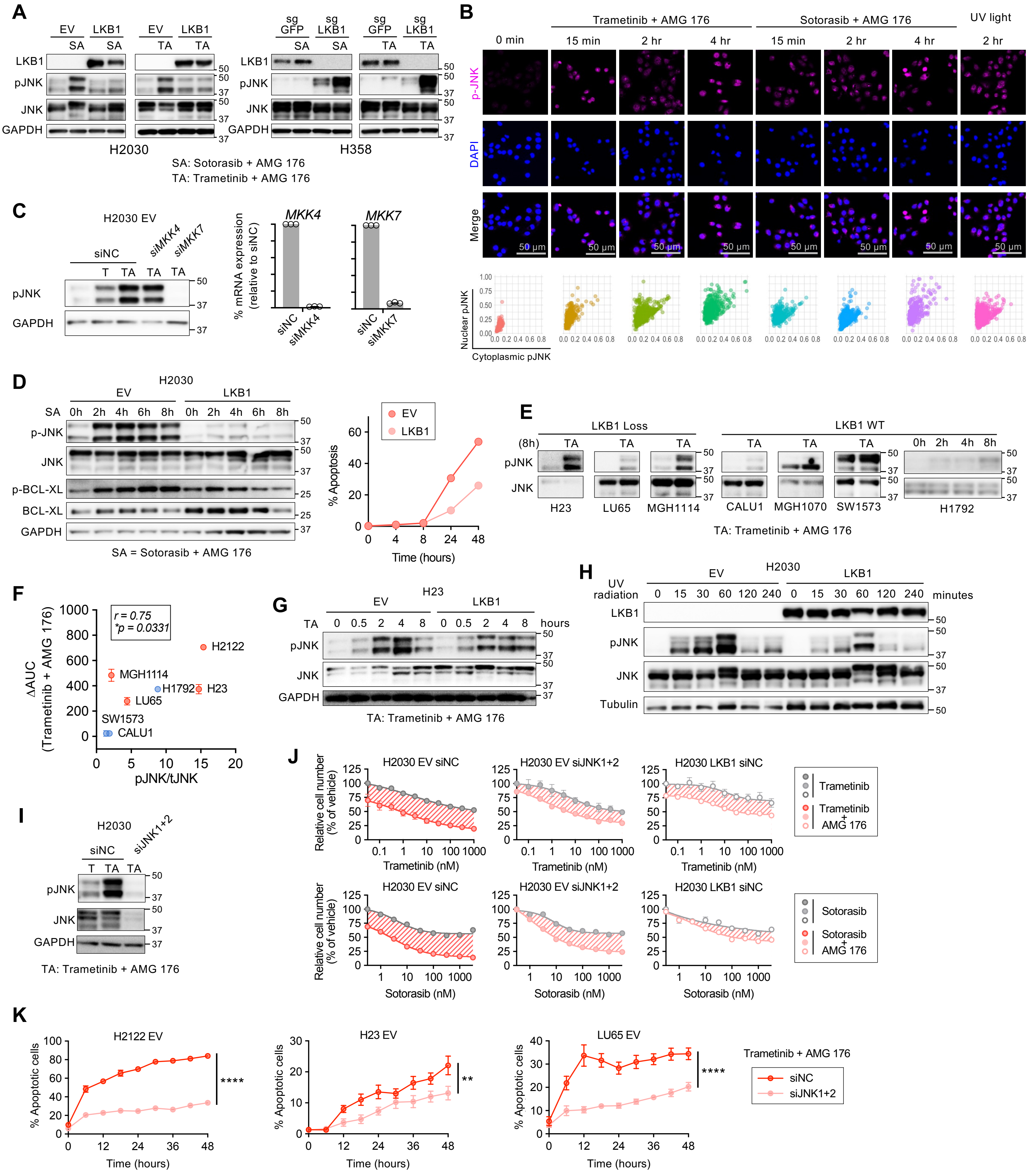

Supplementary Figure 6

Supplementary Figure 6. JNK activation in LKB1-deficient cells underlies MCL-1 dependence. A. Western blot analysis of isogenic *KRAS*-mutant NSCLC cell lines after treatment with 1 µM sotorasib + 1 µM AMG 176 (SA) or 0.1 µM trametinib + 1 µM AMG 176 (TA) for 8 hours. **B.** H2030 cells were treated with sotorasib/trametinib + AMG 176 or UV irradiation for up to 4 hours and phospho-JNK was assessed by immunofluorescence. **C.** Effect of siRNA knock-down of MKK4 and MKK7 on pJNK in H2030 EV cells treated with 0.1 µM trametinib (T) for 24 h or TA. **D.** H2030 isogenic cell lines with EV (empty vector) or LKB1 expression were treated with SA for up to 8 hours and phospho-JNK (left) and annexin staining (right) were performed. **E.** JNK phosphorylation in cell lines treated with TA for 8 hours. **F.** Correlation between phospho-JNK normalized to total JNK v.s. relative sensitivity to TA in cell lines were treated TA for 8 hours. Data are from 3 independent biological replicates, Spearman correlation r=0.75, p=0.0331. **G.** Time-course of JNK phosphorylation in H23 EV and LKB1 cells treated with 0.1 µM trametinib + 1 µM AMG 176. **H.** phospho-JNK in H2030 EV and LKB1 cells after UV irradiation for 0-4 hours. **I.** phospho-JNK in H2030 EV cells with siRNA knockdown of JNK1+2 after treatment with T for 24 hours or TA for 4 hours. **J.** H2030 EV cells with siRNA knockdown of JNK1+2 (or siNC negative control) or H2030 LKB1 cells were treated with trametinib or sotorasib alone or in combination with 1 µM of AMG 176. Cell viability was determined by CTG after 3 days. Data is representative of 3 biological replicates. **K**. H2122, H23, LU65 EV cells with siRNA knockdown of JNK1+2 (or siNC negative control) were treated with TA for 48 hours and apoptosis induction was measured by live-cell imaging. Data are mean and S.E.M. of 3 technical replicates, ****p=0.000001, **p=0.0079, unpaired-nonparametric t test, 2-sided. Source data are provided as a Source Data file.

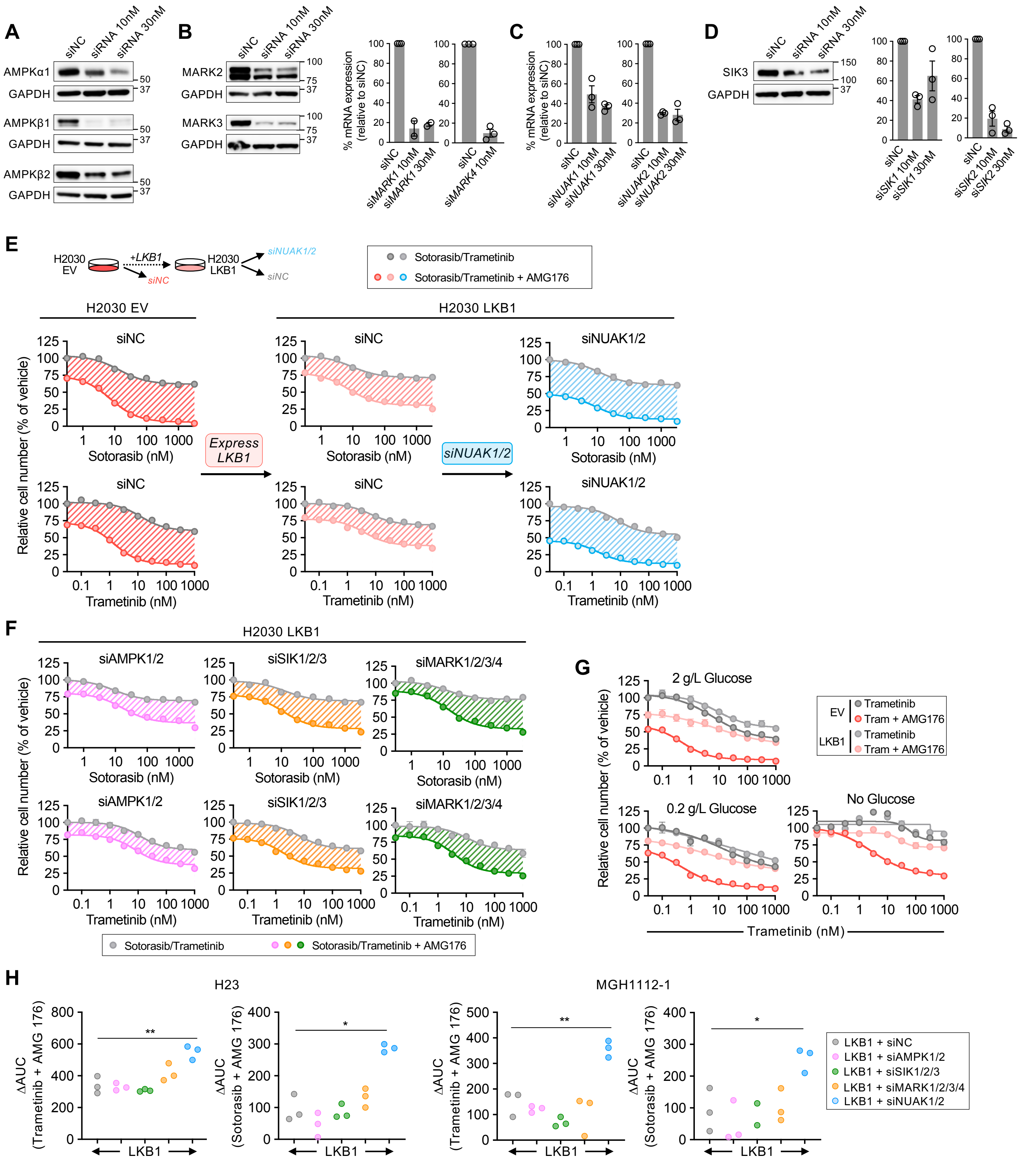

Supplementary Figure 7

Supplementary Figure 7. Suppression of JNK activation by LKB1 is mediated by NUAK kinases. A-D. siRNA knockdown of AMPK-family assessed by western blot or RT-qPCR. **E-F.** Viability assessment H2030 LKB1 cells with corresponding siRNA knockdown after treatment with sotorasib/trametinib alone or in combination with 1 μM AMG 176. Data are representative of 3 biological replicates. **G.** Viability assessment of H2030 EV or LKB1 cells in culture media containing no glucose, 0.2 g/L glucose, or 2 g/L glucose. Data are representative of 3 biological replicates. **H.** Knockdown of NUAK1/2 restores sensitivity (ΔAUC) to combined sotorasib or trametinib + AMG 176. H23 and MGH1112-1 LBK1 cells transfected with corresponding siRNAs were treated with sotorasib or trametinib in the absence or presence of AMG 176 (1 μM) and viability was determined after 3 days. Each dot represents an independent biological replicate (N=3). H23: ***p*=0.0069, **p*=0.0102, MGH1112-1: ***p*=0.0061, **p*=0.0336, unpaired-parametric t test, 2-sided. Source data are provided as a Source Data file. Representative data for western blots has been replicated at least 2 times.

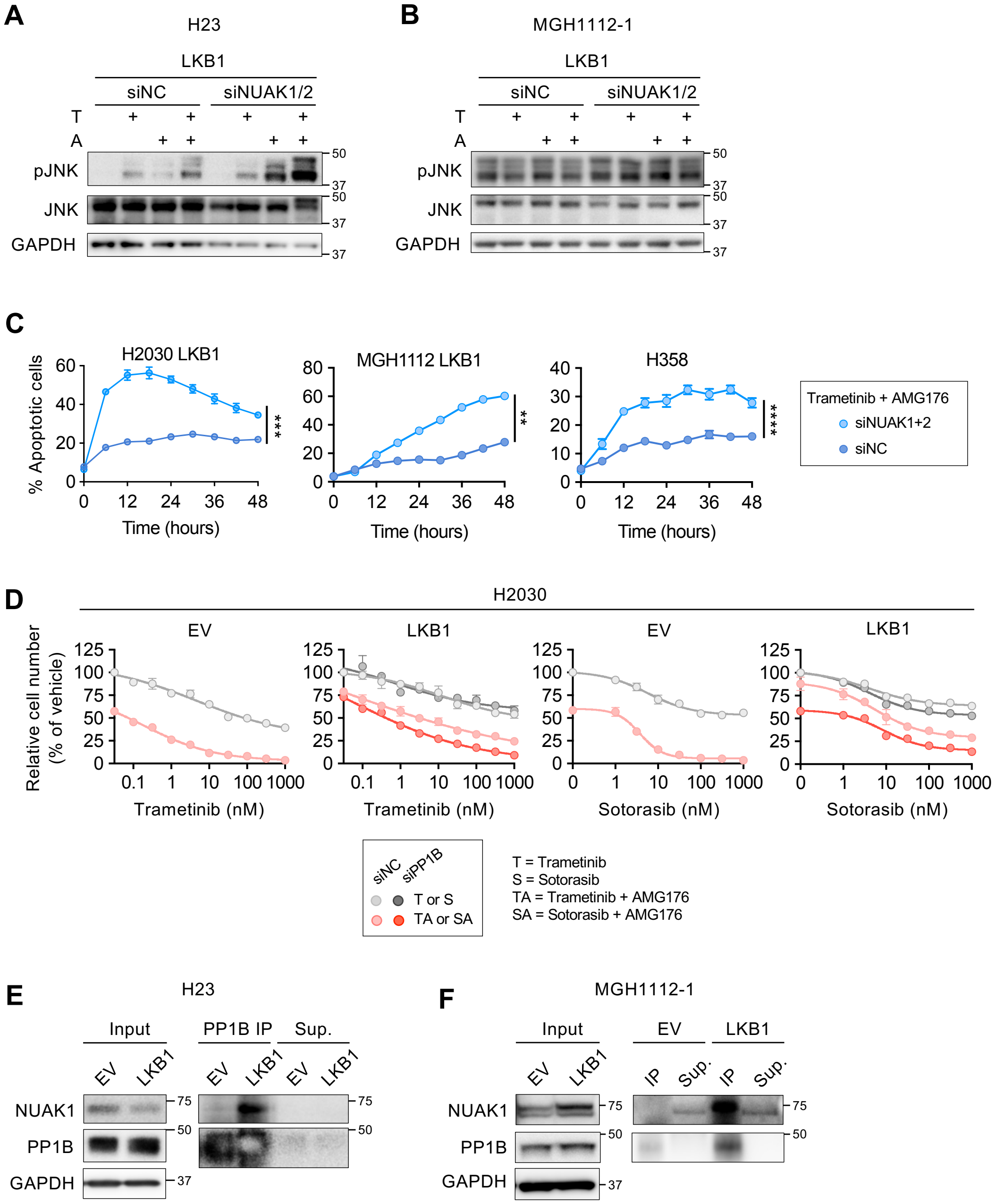

Supplementary Figure 8

Supplementary Figure 8. LKB-NUAK-PP1B axis regulates JNK activation and drug sensitivity. A-B. NUAK1/2 knockdown restores phospho-JNK induction after trametinib or trametinib + AMG 176 in H23, MGH1112-1 LKB1 cells. Cells were transfected with the indicated siRNAs and then treated with trametinib (0.1 μM) for 48 hours, AMG 176 for 4h or trametinib (0.1 μM) or sotorasib (1 μM) for 48 hours followed by AMG 176 for 4 hours. **C.** LKB1-reconstituted (H2030, MGH1112-1) or LKB1 WT (H358) with siRNA knockdown of NUAK1/2 were treated with trametinib + AMG 176 and apoptosis (annexin positivity) was measured by live cell imaging. Data are mean and S.E.M. of 3 technical replicates. H2030: ***p=0.0002, MGH1112: **p=0.0033, H358:****p=0.0001, unpaired-nonparametric t test, 2-sided. **D.** Viability assessment H2030 EV and LKB1 cells with siRNA knockdown of PP1B after treatment with sotorasib/trametinib alone or in combination with 1 μM AMG 176. Data is representative of 3 biological replicates. **E-F.** Restoration of LKB1 expression induces binding between PPIB and NUAK1. HA-tagged WT NUAK1 were over-expressed in H23, MGH1112-1 isogenic cells and the interaction of NUAK1 and PP1B was assessed by immuno-precipitation. Source data are provided as a Source Data file. Western blots and immunoprecipitation images are representative of at least 2 independent biological replicates.

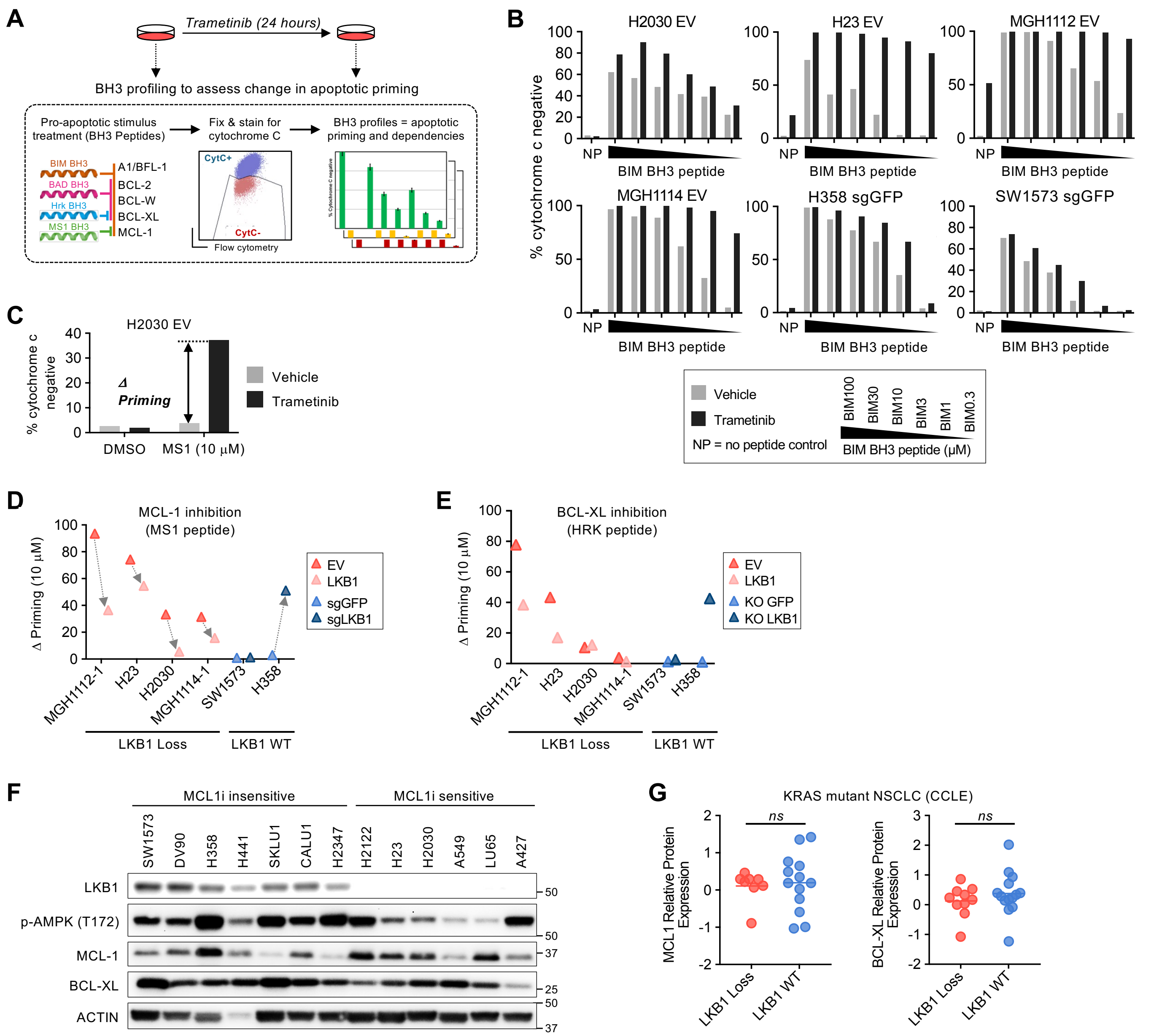

Supplementary Figure 9

Supplementary Figure 9. LKB1 deficiency increases BIM:MCL-1 interaction and creates an MCL-1 dependent state. A. Schematic of BH3 profiling experimental setup. The change in priming (ΔPriming) is measured before and after treatment with trametinib (0.1 μM) as depicted in Figure S9C. **B.** Overall priming of *KRAS*-mutant NSCLC cells treated with vehicle or 0.1 μM of trametinib for 16 hours, as determined by BH3 profiling with titration of BIM BH3 peptide. **C.** Illustration of method for calculating Δpriming (increase or decrease in priming between vehicle and drug treated cells) from BH3 profiling results. **D-E.** Change in MCL-1 or BCL-XL dependence (MS1 BH3 peptide at 30 μM dose, HRK BH3 peptide at 100 μM dose) upon treatment of isogenic cell lines with trametinib. **F.** Expression of MCL-1 and BCL-XL in *KRAS*-mutant NSCLC cell lines grouped according to sensitivity to trametinib/sotorasib + MCL-1 inhibitor. **G.** Relative protein expression level of MCL-1 and BCL-XL in *KRAS*-mutant NSCLC cell lines grouped by LKB1 status from CCLE proteomic database. Source data are provided as a Source Data file. Western blots and immunoprecipitation images are representative of at least 2 independent biological replicates.

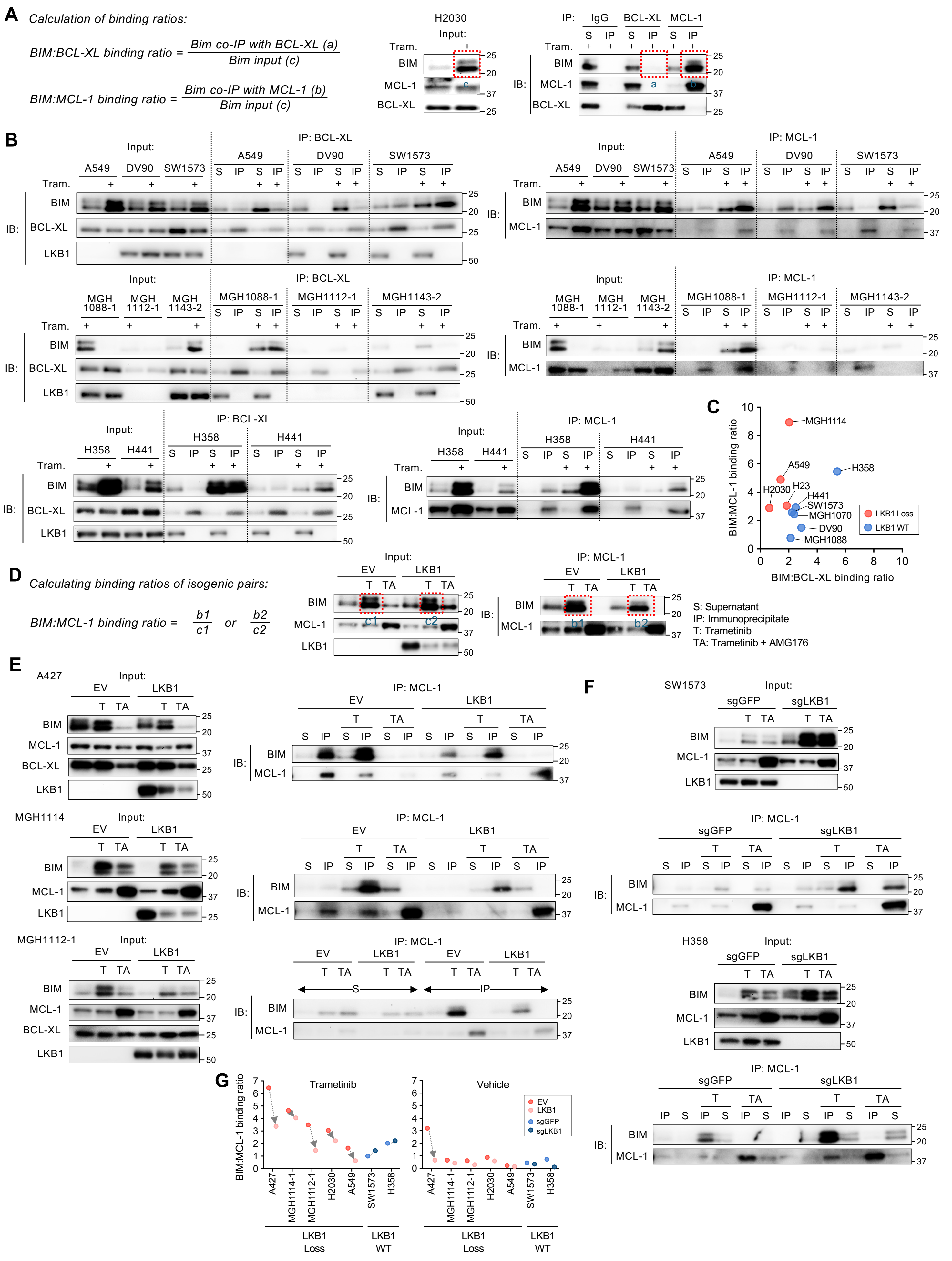

Supplementary Figure 10

Supplementary Figure 10. LKB1 deficiency correlates with BIM:MCL-1 protein binding. A. Method for calculating BIM:MCL-1 and BIM:BCL-XL binding ratios from co-immunoprecipitation (co-IP) of *KRAS*-mutant NSCLC cell lines. Input and IP protein bands were quantified from the same blot membrane. **B.** Co-IP assessment of BIM bound to MCL-1 and BCL-XL binding in *KRAS*-mutant NSCLC cell lines after treatment with 0.1 μM of trametinib for 24 hours. **C.** Quantification of BIM bound to MCL-1 versus BCL-XL in *KRAS*-mutant NSCLC cells after treatment with trametinib. BIM:MCL-1 and BIM:BCL-XL binding ratios were calculated from densitometry measurements as described in Figure S10A. Input and IP protein bands were quantified from the same blot. **D.** Method for calculating BIM: MCL-1 and BIM-BCL-XL binding ratios in isogenic cell lines. Input and IP protein bands were quantified from the same blot membrane. Blot is reprinted from Figure 4A for illustration purposes. **E-F.** Co-IP assessment of BIM bound to MCL-1 in isogenic cell lines after treatment with 0.1 μM of trametinib for 24 hours (T), or 0.1 μM of trametinib for 24 hours + 1 μM of AMG 176 for 4 hours (TA). **G.** BIM:MCL-1 binding ratios after 24 hours trametinib treatment (left) or vehicle (right) in isogenic cell lines. Binding ratios were calculated from densitometry measurements as shown in Figure S10D. EV- empty vector. sgGFP or LKB1 – Crispr KO of GFP or LKB1. Source data are provided as a Source Data file. Representative data for immunoprecipitation has been replicated at least 2 times.

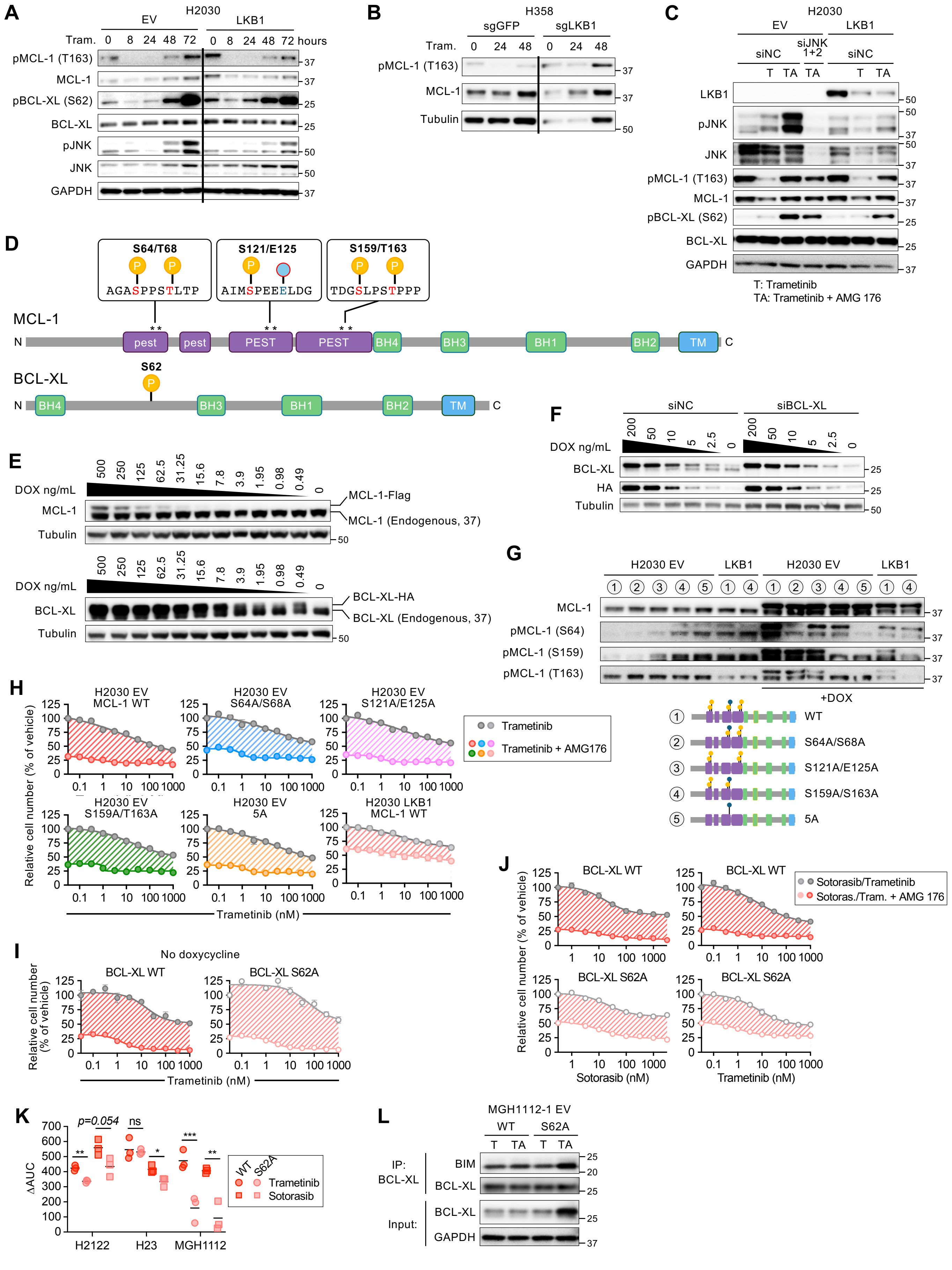

Supplementary Figure 11

Supplementary Figure 11. JNK phosphorylates BCL-XL S62, altering BIM:BCL-XL binding and driving an MCL-1 dependent state. A-B. Time-course of phosphorylation of MCL-1 and BCL-XL in H2030 EV (empty vector) /LKB1 and H358 KO GFP/LKB1 cells treated with 0.1 μM of trametinib. Blots shown in each panel are from a single membrane with non-relevant intervening lanes removed (indicated by solid vertical line). **C.** After siRNA transfection, cells were treated with 0.1 μM trametinib (T) for 48h or trametinib for 48h followed by 1 μM AMG 176 (TA) for 4 hours. Phopho-MCL-1 level were assessed. Figure S6I is duplicated here for comparison purposes. **D.** Schematic of phospho-sites in MCL-1 and BCL-XL phosphorylated by JNK. MCL-1 E125 is a phospho-mimetic site, indicated in red. **E.** Western blot of H2030 EV cells with inducible WT MCL-1-Flag and BCL-XL-HA cultured in media containing various concentrations of doxycycline (DOX). **F.** Western blot of H2030 EV cells with inducible BCL-XL WT and siRNA knockdown of BCL-XL (or negative control) cultured in media containing various concentrations of DOX. **G.** MCL-1 phospho-mutants lack corresponding phosphorylation bands. **H.** Viability assessment of H2030 LKB1 cells with corresponding siRNA knockdown after treatment with sotorasib/trametinib alone or in combination with 1 μM AMG 176. Data are representative of 3 biological replicates. **I.** Viability of H2030 cells reconstituted with DOX-inducible BCL-XL WT or S62A mutants was assessed without DOX. Data are representative of 3 biological replicates + S.E.M. **J.** Viability of H2030 cells reconstituted with DOX- inducible BCL-XL WT or S62A mutant was assessed with optimal DOX concentration to restore endogenous levels as identified in Figure S11E. Data are representative of 3 biological replicates + S.E.M.. **K.** BCL-XL constructs were induced in H2122, H23 or MGH1112-1 cells and sensitivity to trametinib or sotorasib + AMG 176 was measured by CTG. Each dot represents 3 independent biological replicate (H2122: ***p*=0.0016, H23: **p*=0.024, MGH1112: ****p*=0.0072, ***p*=0.0054, unpaired-parametric t test, 2-sided). **L.** Co-IP assessment of BIM bound to BCL-XL WT or S62A in MGH1112 EV cells after treatment with T for 24 hours followed by TA. Source data are provided as a Source Data file.

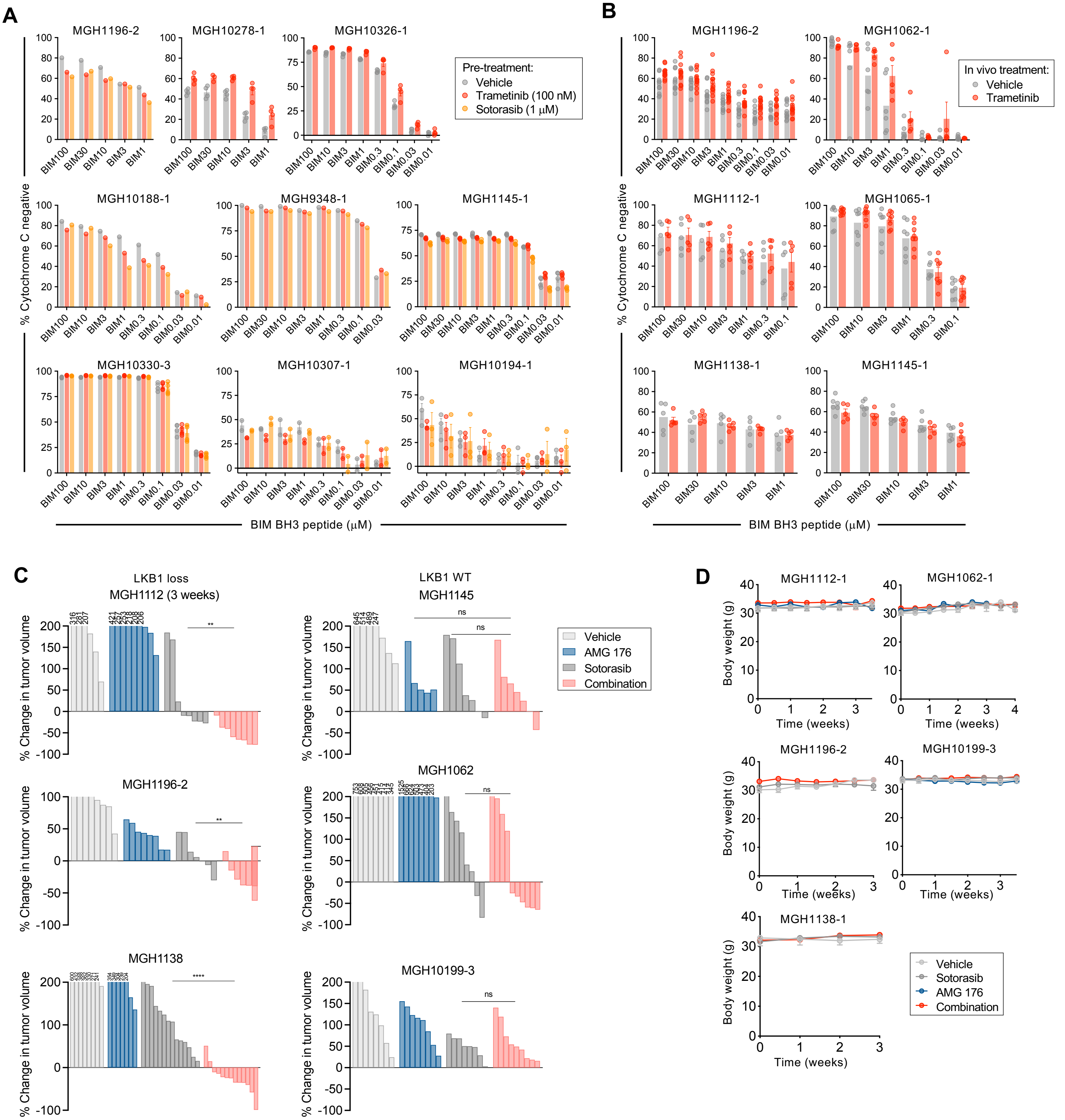

Supplementary Figure 12

Supplementary Figure 12. *Ex vivo* and *in vivo* responses of primary patient and PDX *KRAS*-mutant NSCLCs. A. *KRAS*^{G12C}-mutant NSCLC tumor cells from patient metastatic sites were treated ex vivo with sotorasib or trametinib for 16 hours. Overall priming is shown, as determined by BH3 profiling with titration of BIM BH3 peptide. Change in BIM dependent priming of tumor cells after *ex vivo* treatment with 0.1 μM trametinib or 1 μM sotorasib treatment compared. Each dot represents a technical replicate. **B.** Mice bearing *KRAS*^{G12C}-mutant NSCLC PDX tumors were treated with trametinib (3 mg/kg) for 3 days and harvested for BH3 profiling. Data shown is overall priming as determined by titration of BIM BH3 peptide. Each dot represents an independent tumor, 5-10 animals were used for each treatment group/model. **C.** Waterfall plots showing tumor response of *KRAS*^{G12C}-mutant NSCLC PDX tumors treated with sotorasib (100 mg/kg daily), AMG 176 (50 mg/kg daily), or combination of sotorasib + AMG 176 at indicated time points. MGH1112: ***p*=0.0013, MGH1196-2: ***p*=0.0022, MGH1138: *****p*=0.00001, 2-way ANOVA. **D.** Animal body weights of PDX models treated with sotorasib, AMG176 or combination. 3-8 animals were used for each treatment arm for weight measurement. Source data are provided as a Source Data file.

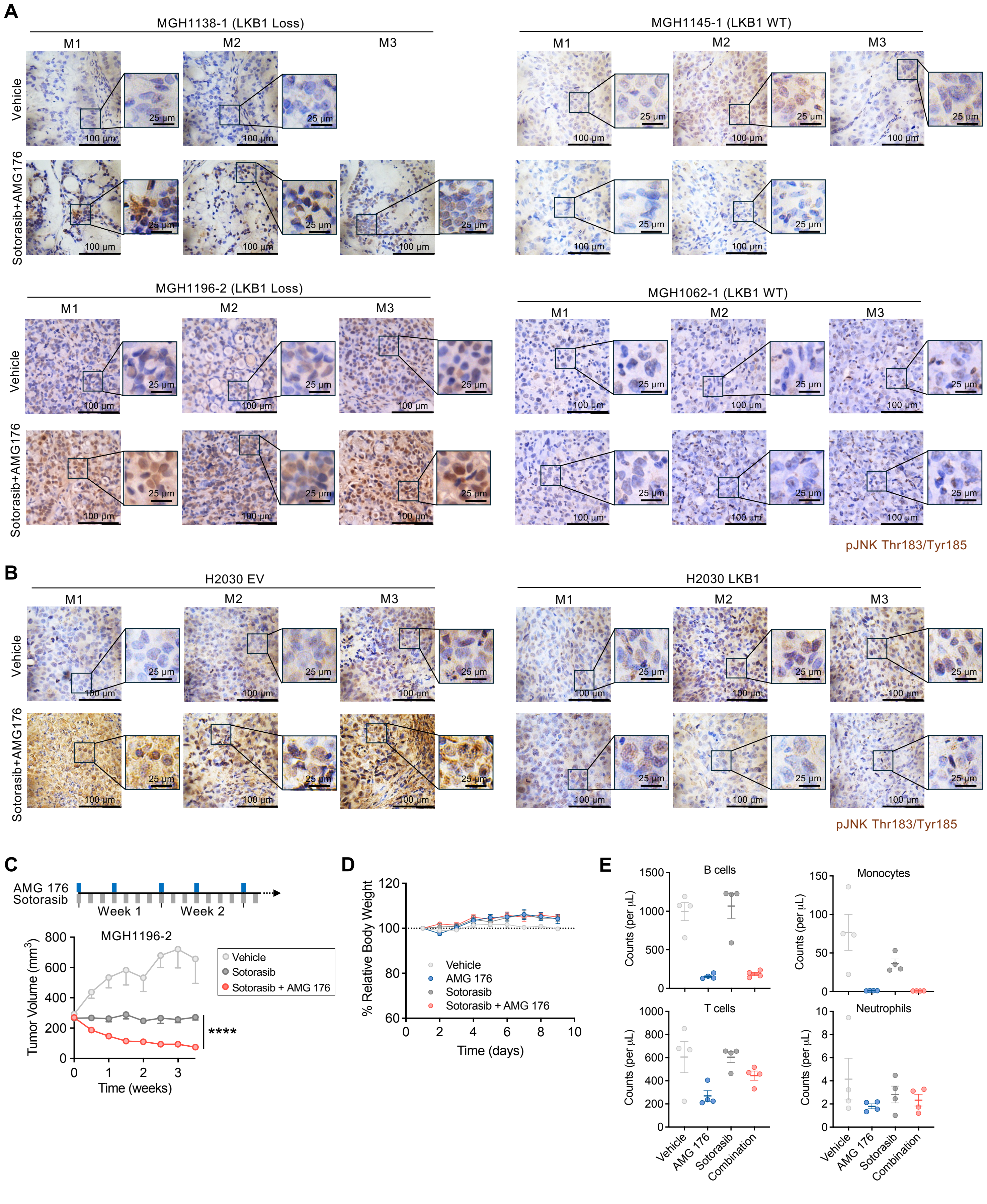

Supplementary Figure 13

Supplementary Figure 13. *In vivo* response of *KRAS*-mutant NSCLC PDX models to sotorasib + AMG 176. A-B. Representative immunohistochemical staining pJNK (Thr183/Tyr185) in MGH1112-1, MGH1145-1, MGH1196-2, MGH1062-1 PDX and H2030 EV (empty vector) and LKB1 xenograft tumors. **C.** Mice bearing MGH1196-2 PDX tumors were treated with sotorasib (100 mg/kg) daily and AMG 176 (50 mg/kg) twice weekly. Tumor response was similar to AMG 176 dosed daily (see Figure 6E). Data are mean and S.E.M., N=5 animals per treatment group/model. ****p=0.0002, 2-way ANOVA. **D-E.** Humanized MCL-1 knock-in mice were treated with sotorasib (100 mg/kg) daily with twice weekly dosing of AMG 176 (50 mg/kg). Body weight was monitored. B cells, T cells, plasma cells, monocytes, and neutrophils from peripheral blood (24 hours after cycle 2) were characterized by flow cytometry. Each dot corresponds to an individual mouse (N=4 per treatment group). Source data are provided as a Source Data file. Western blots and immunoprecipitation images are representative of at least 2 independent biological replicates.
