# Supplementary material for: LKB1 regulates JNK-dependent stress signaling and apoptotic dependency of KRAS-mutant lung cancers
Source: Nat Commun. 2025 May 2;16:4112. doi: 10.1038/s41467-025-58753-y (PMC12048556; doi:10.1038/s41467-025-58753-y)
Supplement: Supplementary file 2 — Description of Additional Supplementary Information [file 41467_2025_58753_MOESM2_ESM.docx]

**Description of Additional Supplementary Files**

File Name: Supplementary Data 1

Description: IC50 and EMAX values for sotorasib in KRAS-mutant NSCLC cell lines.

File Name: Supplementary Data 2

Description: RT-PCR primer and sgRNA sequences used in this study.
