# Supplementary material for: LKB1 regulates JNK-dependent stress signaling and apoptotic dependency of KRAS-mutant lung cancers
Source: Nat Commun. 2025 May 2;16:4112. doi: 10.1038/s41467-025-58753-y (PMC12048556; doi:10.1038/s41467-025-58753-y)
Supplement: Supplementary file 5 — Reporting Summary [file 41467_2025_58753_MOESM5_ESM.pdf]

nature portfolio

Corresponding author(s): Aaron N. Hata

Last updated by author(s): Mar 15, 2025

Reporting Summary

Nature Portfolio wishes to improve the reproducibility of the work that we publish. This form provides structure for consistency and transparency in reporting. For further information on Nature Portfolio policies, see our Editorial Policies and the Editorial Policy Checklist.

Statistics

For all statistical analyses, confirm that the following items are present in the figure legend, table legend, main text, or Methods section.

n/a	Confirmed	
☐	☒	The exact sample size (*n*) for each experimental group/condition, given as a discrete number and unit of measurement
☐	☒	A statement on whether measurements were taken from distinct samples or whether the same sample was measured repeatedly
☐	☒	The statistical test(s) used AND whether they are one- or two-sided<br>*Only common tests should be described solely by name; describe more complex techniques in the Methods section.*
☒	☐	A description of all covariates tested
☒	☐	A description of any assumptions or corrections, such as tests of normality and adjustment for multiple comparisons
☐	☒	A full description of the statistical parameters including central tendency (e.g. means) or other basic estimates (e.g. regression coefficient) AND variation (e.g. standard deviation) or associated estimates of uncertainty (e.g. confidence intervals)
☐	☒	For null hypothesis testing, the test statistic (e.g. *F*, *t*, *r*) with confidence intervals, effect sizes, degrees of freedom and *P* value noted<br>*Give P values as exact values whenever suitable.*
☐	☒	For Bayesian analysis, information on the choice of priors and Markov chain Monte Carlo settings
☐	☒	For hierarchical and complex designs, identification of the appropriate level for tests and full reporting of outcomes
☐	☒	Estimates of effect sizes (e.g. Cohen's *d*, Pearson's *r*), indicating how they were calculated

Our web collection on statistics for biologists contains articles on many of the points above.

Software and code

Policy information about availability of computer code

Data collection	No software was used.
Data analysis	Phospho-proteomic data analysis: sequest algorithm (PMID: 24226387); in house-built platform (PMID: 21183079); quantification (PMID: 21963607). Phospho-proteomic signature analysis: PTM-Signature Enrichment Analysis (PMT-SEA)(https://github.com/broadinstitute/ssGSEA2.0). Synergy analysis was performed using Biochemically Intuitive Generalized Loewe (BIGL)

For manuscripts utilizing custom algorithms or software that are central to the research but not yet described in published literature, software must be made available to editors and reviewers. We strongly encourage code deposition in a community repository (e.g. GitHub). See the Nature Portfolio guidelines for submitting code & software for further information.

Data

Policy information about availability of data

All manuscripts must include a data availability statement. This statement should provide the following information, where applicable:

- Accession codes, unique identifiers, or web links for publicly available datasets
- A description of any restrictions on data availability
- For clinical datasets or third party data, please ensure that the statement adheres to our policy

Raw phosphoproteomic data generated in this study have been deposited in MassIVE [https://massive.ucsd.edu/ProteoSAFe/static/massive.jsp] under accession code “MSV000097246”, as well as Proteomexchange [https://www.proteomexchange.org/] under accession code “PXD061550, doi:10.25345/C5HH6CJ48”. Processed phophoproteomic data (normalized intensity) can be downloaded from Harvard Dataverse using identifier “https://doi.org/10.7910/DVN/OLVIT7”. All the

other raw data, including graphs and western blots, are provided in the Supplementary Information/Source Data file. All flow cytometry gating strategies are provided in the Supplementary Information/FACs Gating file.

Research involving human participants, their data, or biological material

Policy information about studies with human participants or human data. See also policy information about sex, gender (identity/presentation), and sexual orientation and race, ethnicity and racism.

Reporting on sex and gender	Patients were chosen based on KRAS and/or STK11 somatic mutation. There was no discrimination based on gender.
Reporting on race, ethnicity, or other socially relevant groupings	Patients were chosen based on KRAS and/or STK11 somatic mutation. There was no discrimination based on race, ethnicity, or other socially relevant groupings
Population characteristics	Patients were chosen based on KRAS and/or STK11 somatic mutation.
Recruitment	Patients were chosen based on KRAS and/or STK11 somatic mutation.
Ethics oversight	Tumors or pleural fluid were collected IRB protocol at Massachusetts General Hospital. The cell line and xenograft development was performed under IRB protocol at Dana Farber/ Harvard cancer center.

Note that full information on the approval of the study protocol must also be provided in the manuscript.

Field-specific reporting

Please select the one below that is the best fit for your research. If you are not sure, read the appropriate sections before making your selection.

[x] Life sciences [ ] Behavioural & social sciences [ ] Ecological, evolutionary & environmental sciences

For a reference copy of the document with all sections, see nature.com/documents/nr-reporting-summary-flat.pdf

Life sciences study design

All studies must disclose on these points even when the disclosure is negative.

Sample size	For the in vitro study screening a panel of cell lines, we included all commercially available and MGH-derived patient cell lines (100%). For the in vitro study assessing whether LKB1 can manipulate sensitivity, we included 40% of the cell lines to represent the population. For in vitro cell line biological replicates, we used at least three replicates to perform a Student's t-test. Each in vitro cell line experiment included at least three biological replicates. For the in vivo study, we used 4–7 animals per arm to perform a Student's t-test or 2-way ANOVA.
Data exclusions	No data was excluded.
Replication	For all in vitro and in vivo data, 7-10 biological replicates were performed and consistent results were observed.
Randomization	In the in vivo study, mice were assigned randomly to each treatment group.
Blinding	The investigators were not blinded during the experiments and out assessments, because the same investigators were needed to perform the treatment, measurement and monitoring.

Reporting for specific materials, systems and methods

We require information from authors about some types of materials, experimental systems and methods used in many studies. Here, indicate whether each material, system or method listed is relevant to your study. If you are not sure if a list item applies to your research, read the appropriate section before selecting a response.

Materials & experimental systems

n/a	Involved in the study	
[ ]	[x]	Antibodies
[ ]	[x]	Eukaryotic cell lines
[x]	[ ]	Palaeontology and archaeology
[ ]	[x]	Animals and other organisms
[x]	[ ]	Clinical data
[x]	[ ]	Dual use research of concern
[x]	[ ]	Plants

Methods

n/a	Involved in the study	
[x]	[ ]	ChIP-seq
[ ]	[x]	Flow cytometry
[x]	[ ]	MRI-based neuroimaging

Antibodies

Antibodies used	pJNK T183/Y185 (CST4668), SAPK/JNK (CST9252), BIM (CST2933), pBCL-XL S62 (Invitrogen 44-428G), BCL-XL (CST2764), LKB1 (CST3050), pMCL-1 T163 (CST14765), pMCL-1 S159/T163 (CST4579), pMCL-1 S64 (CST13297), MCL-1 (BD Pharmingen 559027), pMKK4 S257/T261 (CST9156), MKK4 (CST9152), pMEK7 S271 (Thermo Fisher PA5-114604), pMEK7 T275 (Thermo Fisher PA5-114605), MKK7 (CST4172), DUSP10/MKP5 (CST3483), HA Tag (CST3724), β-Tubulin (CST2146), GAPDH (CST5174). We used a 1:1000 dilution for all Western blot analyses and a 1:200 dilution for immunofluorescence and immunohistochemistry.
Validation	pJNK T183/Y185, SAPK/JNK, LKB1, pMKK4, MKK4, pMEK7, pMEK7 T275, MKK7, BCL-XL, MCL-1, and DUSP10/MKP5 antibody was validated by removing the protein by either siRNA mediated gene silencing or Crispr mediated gene knock out, data was included in the manuscript. Bim, β-Tubulin and GAPDH were validated in previous published study (PMID: 30254092). pBCL-XL S62, pMCL-1 T163, pMCL-1 S159/T163 and pMCL-1 S64 were validated by DOX-inducible BCL-XL or MCL-1 mutant cell lines, data was included in the manuscript.

Eukaryotic cell lines

Policy information about cell lines and Sex and Gender in Research

Cell line source(s)	Publicly available KRAS-mutant NSCLC cell lines were obtained from the Center for Molecular Therapeutics at the Massachusetts General Hospital (MGH) Cancer Center. Patient-derived NSCLC cell lines were established in our laboratory from surgical resections, core-needle biopsies, or pleural effusion samples, as previously described (PMID: 30254092), except for the MGH1070 cell line, which was derived from a primary mouse PDX model. All patients provided informed consent to participate in a Dana-Farber/Harvard Cancer Center Institutional Review Board–approved protocol, granting permission for research to be conducted on their samples.
Authentication	STR validation was performed at the initiation of the project (Biosynthesis, Inc.).
Mycoplasma contamination	Cell lines were routinely tested for mycoplasma during experimental use.
Commonly misidentified lines (See ICLAC register)	No commonly misidentified cell lines were used.

Animals and other research organisms

Policy information about studies involving animals; ARRIVE guidelines recommended for reporting animal research, and Sex and Gender in Research

Laboratory animals	NSG or athymic nude (NE/Nu) were purchased from Jackson Laboratory with age 8-12 weeks.
Wild animals	The study did not involved wild animals.
Reporting on sex	For nude mice, female mice were used. For NSG mice, only male mice were used due to high aggressiveness of the female mice.
Field-collected samples	No field-collected samples.
Ethics oversight	All animal studies were conducted through MGH Institutional Animal Care and Use Committee (IACUC) –approved animal protocols in accordance with institutional guidelines. (Protocol number 2008N000095, 2008N000054)

Note that full information on the approval of the study protocol must also be provided in the manuscript.

Plants

Seed stocks	No plant seeds were used in this study
Novel plant genotypes	Not applicable
Authentication	Not applicable

Flow Cytometry

Plots

Confirm that:

- [x] The axis labels state the marker and fluorochrome used (e.g. CD4-FITC).
- [x] The axis scales are clearly visible. Include numbers along axes only for bottom left plot of group (a 'group' is an analysis of identical markers).
- [x] All plots are contour plots with outliers or pseudocolor plots.
- [x] A numerical value for number of cells or percentage (with statistics) is provided.

Methodology

Sample preparation	Cells were seeded in triplicate at low density 24 hours prior to drug addition. Seventy-two hours after adding drugs, floating (dead) and adherent cells (alive) were collected and stained with propidium iodide (PI) and Cy5-Annexin V (BD Biosciences)
Instrument	BD LSRFortessa
Software	Data was collected by FACSDiva and analyzed by FlowJo.
Cell population abundance	Annexin-positive apoptotic cell fraction ranges from 3%-90% based on the treatment arms.
Gating strategy	Positive gates (dead cell) were defined by cells treated with EtOH.

- [x] Tick this box to confirm that a figure exemplifying the gating strategy is provided in the Supplementary Information.
